# Physicians’ Mutual Benefit Association

**Published:** 1885-10

**Authors:** W. A. Morris, F. E. Daniel

**Affiliations:** President, Austin, Texas; Secretary and Treasurer, Austin, Texas


### TO THE PHYSICIANS OF TEXAS.

AUSTIN, TEXAS, October 10, 1885.

*Dear Doctor:*

THE Physicians' Mutual Benefit Association of Texas was chartered by the State April 25, 1884. It is a purely charitable organization, the object being to provide a fund for the widow and children of deceased members. It is mutual insurance on the least expensive plan ever devised, as there are no salaried officers and no heavy expenses. Membership entails no trouble, no loss of time, no duties to be performed, and *no expense*, or next to none. The Association is subject to no tax. The *only condition to membership* is a *promise* that when a member dies each of the others will contribute one dollar to a fund for the benefit of the widow and children. Unlike other insurance, the assessments are not large in proportion to age, but uniformly one dollar, no more, no less. On the death of a member, each survivor is called on for one dollar, and the aggregate constitutes the amount of the benefit. Thus it is mutual. There is no physician worthy the name, we hope, who would not cheerfully give a deceased brother's family one dollar if called on to do so. This organization is only an arrangement whereby it is made somebody's business to get together those dollars and place them in the hands of the beneficiary. There are only sixty-eight members. There should be one thousand. There has been no death in the membership up to date. If we had five hundred members, on the death of one, to be able to send his family four hundred and ninety-nine dollars would be a real charity; and yet it would cost each survivor only one dollar—the amount he earns for writing a single office prescription! Were the membership one thousand, the deaths (according to statistics of *all classes*) would be only ten annually; but in a single class—a class whose average life is above many other callings—it is not probable the number of deaths would exceed five a year. Thus a benefit of nine hundred and ninety-nine dollars (insurance policy) would cost only six dollars a year—certainly the cheapest insurance possible. The only expense attending membership is one dollar, paid on becoming a member, and annually thereafter on the first of January. This creates a fund for expenses—printing, stationery, books, postage, office rent, etc.—and, if any is left, to pay the secretary for his trouble. We desire to secure the co-operation of town and county societies in this noble work, and will be glad to distribute blank applications for membership. Certificates of membership are issued in lieu of policies.

Yours fraternally,

W. A. Morris, M. D., President, Austin.

F. E. Daniel, M. D., Secretary and Treasurer, Austin.

---

We are authorized to announce that Dr. J. W. Garnett, of Greenville, Hunt county, has been appointed secretary of the Section of Obstetrics, T. S. M. A. Dr. C. C. Francis, of Cleburne, is chairman. Dr. J. W. Daniel, of Houston, has been appointed secretary of the Section on Dermatology. These appointments were too late for the printed Transactions.

